# Correction: Exosome component 1 cleaves single-stranded DNA and sensitizes human kidney renal clear cell carcinoma cells to poly(ADP-ribose) polymerase inhibitor

**DOI:** 10.7554/eLife.112212

**Published:** 2026-06-02

**Authors:** Qiaoling Liu, Qi Xiao, Zhen Sun, Bo Wang, Lina Wang, Na Wang, Kai Wang, Chengli Song, Qingkai Yang

**Keywords:** Mouse

Liu Q, Xiao Q, Sun Z, Wang B, Wang L, Wang N, Wang K, Song C, Yang Q. 2021. Exosome component 1 cleaves single-stranded DNA and sensitizes human kidney renal clear cell carcinoma cells to poly(ADP-ribose) polymerase inhibitor. *eLife* **10**:e69454. doi: 10.7554/eLife.69454.

Published 23 June 2021

Following post-publication review of our manuscript, we identified errors in the manuscript.

In Figure 1—figure supplement 1A, the top-ranked genes of each substitution type (Supplementary file 1) were used for Gene Ontology (GO) enrichment analysis with Metascape (metascape.org). During data processing, the data corresponding to the C>T/G>A substitution were misapplied to the C>G/G>C group. The C>T/G>A mutation should be shown to be enriched in GO:0140053 mitochondrial gene expression, GO:0000959, GO:0007005, GO:1902775 and GO:0034620. We have updated Figure 1—figure supplement 1A and source data accordingly. Because the findings and conclusions were based on the original data, these corrections do not affect the conclusion that substitution mutations were enriched in ‘mitochondrial gene expression’.

In Figure 2J and Figure 2—figure supplement 1D, the labels for the A>G/T>C and A>C/T>G mutations were inadvertently reversed. In addition, the mutation frequency of A>G/T>C should be 3.13% but not 31.3%. The figures and source data have been corrected accordingly. These corrections do not affect the conclusion for C>A/G>T mutation.

In Figure 2B-2D and Figure 5E, illustrative images based on the incomplete experiments were mistaken as final experimental images. During the handover of research work, illustrative images based on incomplete experiments were applied to show the study information and figure layout. Due to a miscommunication between the authors, the illustrative images (EXOSC1 and several groups in Figure 2B-2D, and TUHR14TKB cells in Figure 5E) were mistaken as final images for publication. To rectify these issues, the authors reviewed all original data and experimental records. The incorrect panels and source data have been replaced with the appropriate ones.

The authors deeply apologize to the editors, reviewers, and the scientific community for the errors. The correction did not affect the conclusion. No corrections to the text were needed.

The corrected Figure 1—figure supplement 1 (updated for panel A) is shown here:

**Figure 1:**
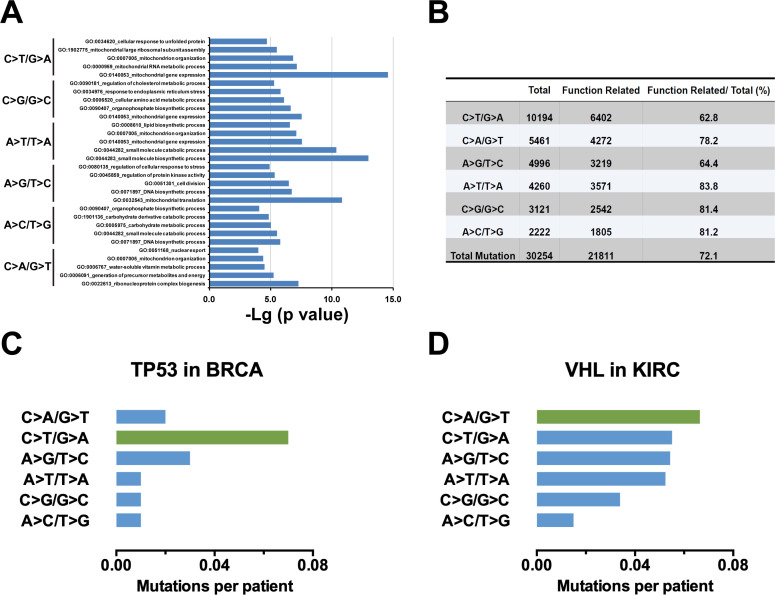


The originally published Figure 1—figure supplement 1 is shown for reference:

**Figure 2:**
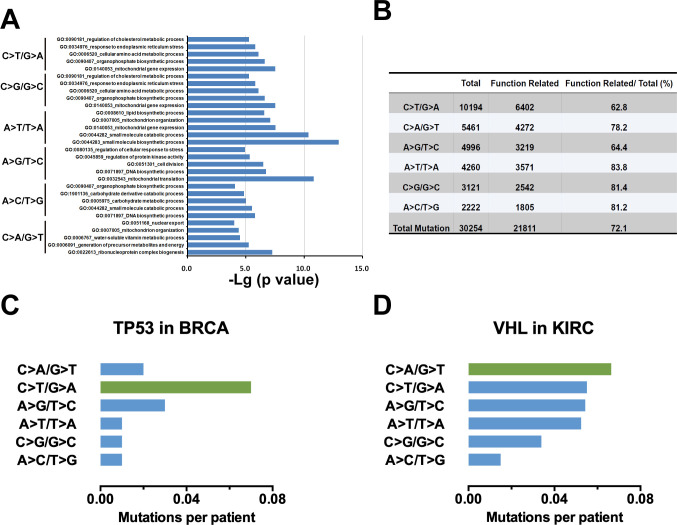


The corrected Figure 2 (updated for panels B, C, D and J) is shown here:

**Figure 3:**
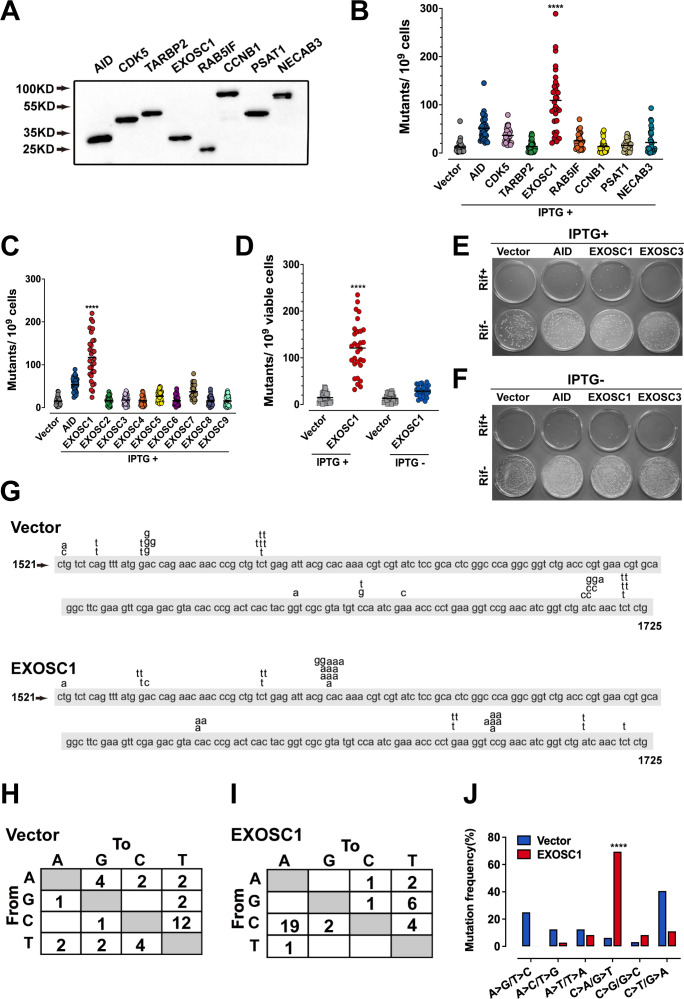


The originally published Figure 2 is shown for reference:

**Figure 4:**
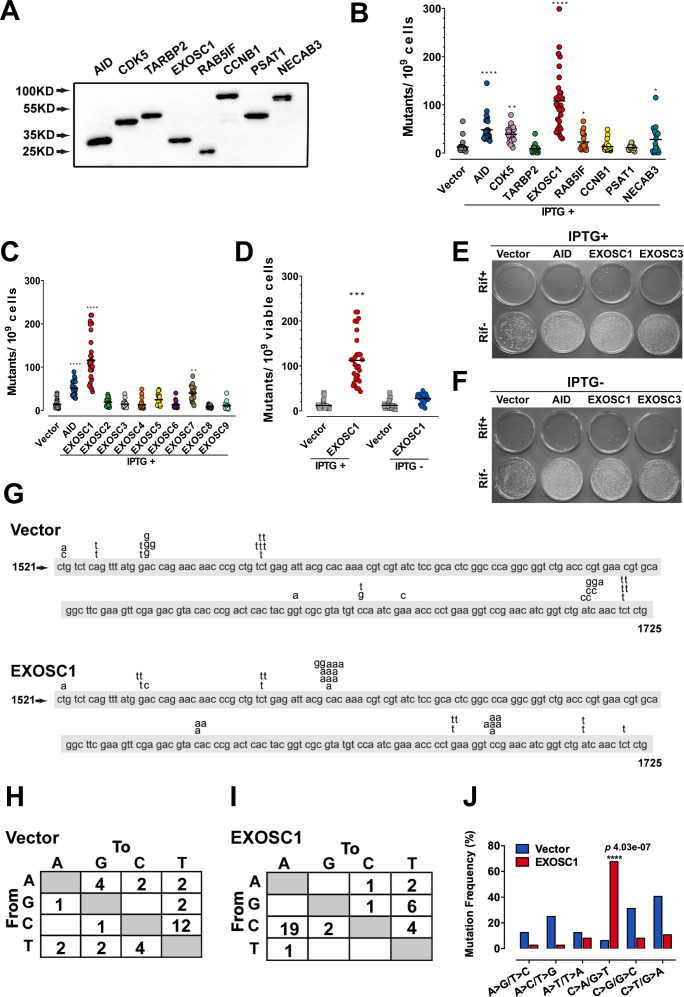


The corrected Figure 2—figure supplement 1 (updated for panel D) is shown here:

**Figure 5:**
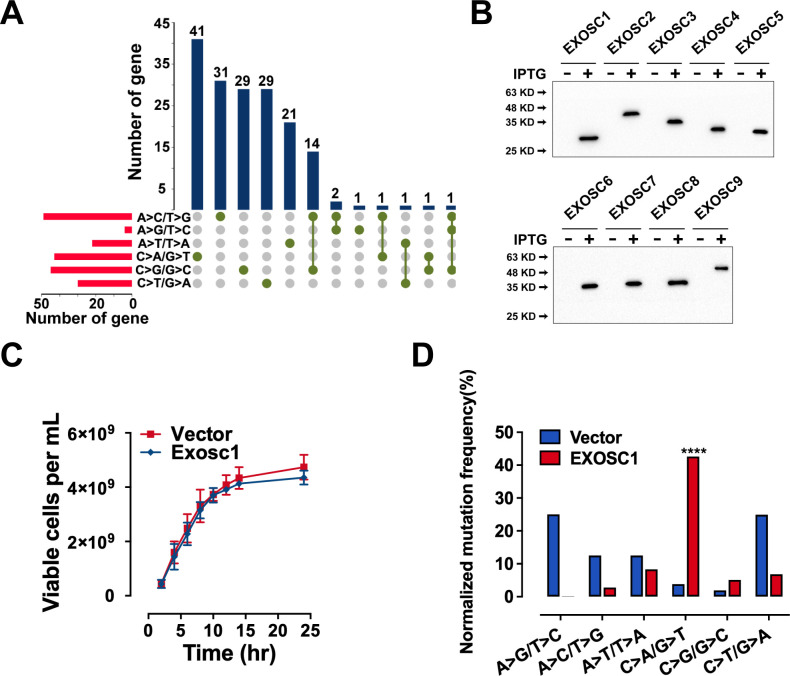


The originally published Figure 2—figure supplement 1 is shown for reference:

**Figure 6:**
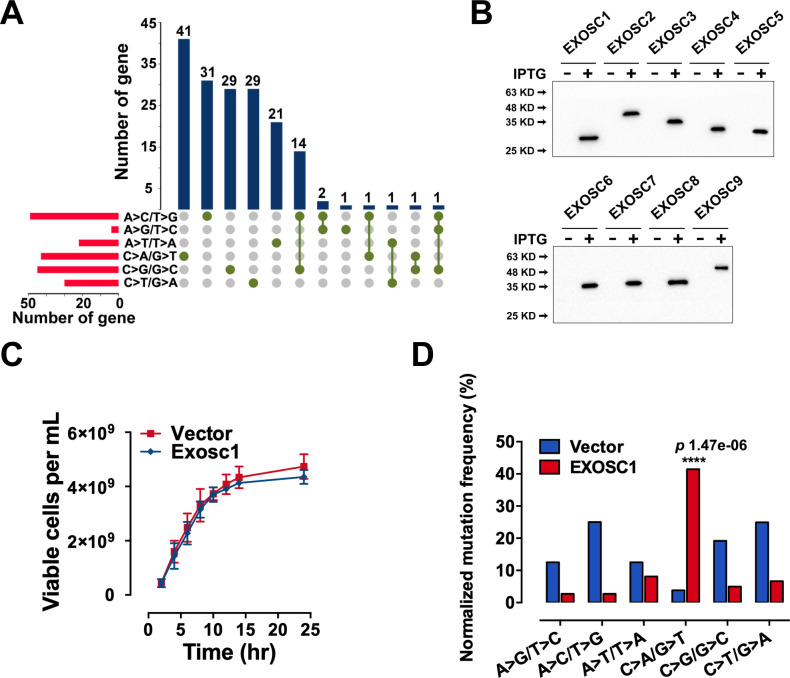


The corrected Figure 5 (updated for panel E) is shown here:

**Figure 7:**
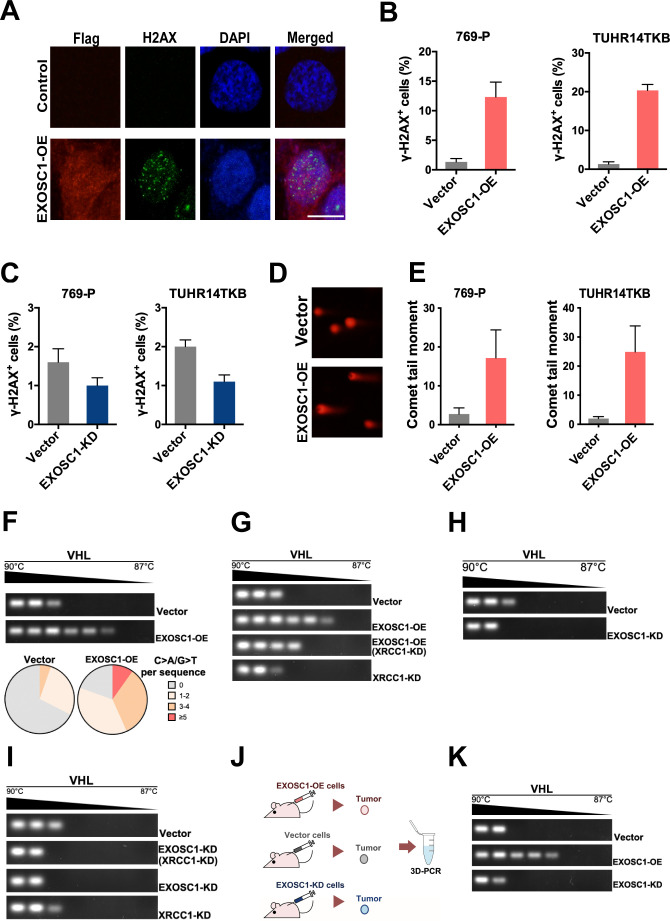


The originally published Figure 5 is shown for reference:

**Figure 8:**
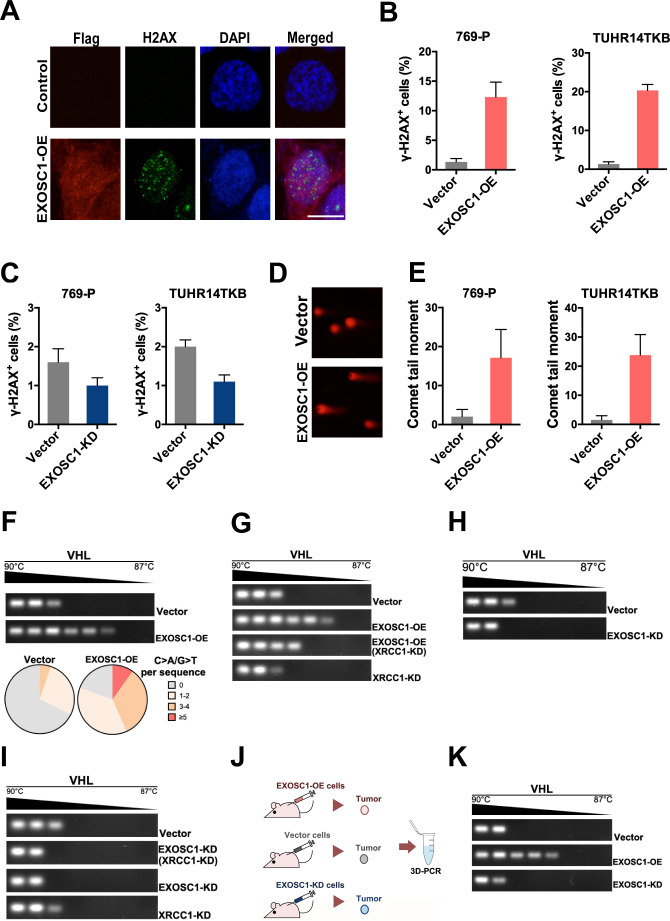


The article has been corrected accordingly.

